# Gram-negative bacterial sRNAs encapsulated in OMVs: an emerging class of therapeutic targets in diseases

**DOI:** 10.3389/fcimb.2023.1305510

**Published:** 2024-01-30

**Authors:** Mobarakeh Ajam-Hosseini, Fatemeh Akhoondi, Farshid Parvini, Hossein Fahimi

**Affiliations:** ^1^ Department of Molecular Genetics, Faculty of Biological Sciences, Tarbiat Modares University, Tehran, Iran; ^2^ Department of Molecular Biology of The Cell, Faculty of Bioscience, University of Milan, Milan, Italy; ^3^ Department of Biology, Faculty of Basic Sciences, Semnan University, Semnan, Iran; ^4^ Department of Genetics, Faculty of Advanced Science and Technology, Tehran Medical Sciences, Islamic Azad University, Tehran, Iran

**Keywords:** gram-negative bacteria, sRNA, extracellular vesicles, therapeutics, diseases

## Abstract

Small regulatory RNAs (sRNAs) encapsulated in outer membrane vesicles (OMVs) are critical post-transcriptional regulators of gene expression in prokaryotic and eukaryotic organisms. OMVs are small spherical structures released by Gram-negative bacteria that serve as important vehicles for intercellular communication and can also play an important role in bacterial virulence and host-pathogen interactions. These molecules can interact with mRNAs or proteins and affect various cellular functions and physiological processes in the producing bacteria. This review aims to provide insight into the current understanding of sRNA localization to OMVs in Gram-negative bacteria and highlights the identification, characterization and functional implications of these encapsulated sRNAs. By examining the research gaps in this field, we aim to inspire further exploration and progress in investigating the potential therapeutic applications of OMV-encapsulated sRNAs in various diseases.

## 1 Introduction

Since the identification of bacteria as the primary cause of infectious diseases, researchers have been interested in the variety of interactions between these tiny organisms and their environment. In particular, bacterial interaction with eukaryotic host cells has gained significant attention during the past few decades (Kaparakis-Liaskos and Kufer, 2020). Bacterial cells release membrane vesicles known as extracellular vesicles (EV) to communicate with host cells and other bacteria (Hosseini-Giv et al., 2022). Depending on the cell structure, Gram-positive and Gram-negative bacteria use various ways to create extracellular vesicles, known as outer membrane vesicles (OMVs) and membrane vesicles (MVs), respectively (Bose et al., 2021). OMVs were first discovered in *Escherichia coli* (*E. coli*) in the 1960s, however, Gram-positive MVs were discovered much later because scientists believed that the thick cell wall that surrounds these bacteria would prevent the release of MVs (Brown et al., 2015; Srivastava and Kim, 2022; Huang et al., 2023). The first proof of vesiculation in Gram-positive bacteria was presented by Dorward and Garon in 1990 (Bose et al., 2021).

EVs are small biological structures that are released by cells that in both in physiological and pathological conditions, can play a central role in cell-cell communication and transfer a variety of cargos, including lipids, proteins, and nucleic acids, which are employed to interact with and have an impact on host cells, such as cytotoxicity and immunomodulation (Hosseini-Giv et al., 2022; Palazzolo et al., 2022). EVs have also been used as drug delivery systems since they have characteristics that made them ideal for this purpose, leading to interesting results in preclinical and clinical trials (Palazzolo et al., 2022). When EVs enter target cells, they release their contents, which include proteins, short RNAs (sRNAs), tRNA fragments, and microRNAs (miRNAs), which then control the gene expression and function of the recipient cell (Stanton, 2021). Recent studies have demonstrated that bacterial vesicles containing noncoding regulatory RNAs are released into the surrounding environment, and transferred to other microorganisms and host cells, as already reported by the protozoan pathogen *Trypanosoma cruzi*. However, intracellular bacterial pathogens can express sRNAs that have regulatory functions in a similar manner as miRNAs. The significance of microbial sRNAs as molecules that can mediate host-microbe interactions is highlighted by recent studies which have demonstrated that bacterial vesicles containing noncoding regulatory RNAs are released into the surrounding environment, and transferred to other microorganisms and host cells, as already reported for the protozoan pathogen Trypanosoma cruzi. However, intracellular bacterial pathogens can also express sRNAs that have regulatory functions in a similar manner as miRNAs (Ahmadi Badi et al., 2020; Stanton, 2021). Extracellular sRNAs can then be found in a variety of bodily fluids, such as serum, plasma, and urine, and they exhibit altered circulating levels in a wide range of disorders, making them potential biomarker possibilities for pathological states (Cucher et al., 2023).

EVs secreted by the microbiota have recently emerged as a new means of communication. The largest microbial ecosystem in the human body, the gut microbiota, carry the message of antibiotic resistance to the surrounding bacteria. Furthermore, they function as a powerful system for the detoxification of substances that are harmful to bacterial growth (Heydari et al., 2022). In the intercellular signaling system, MEVs have become important mediators that may play a crucial role in the communication between the microbiota and the host. Microbiota-derived EVs (MEVs) are tiny membrane-bound vesicles that contain a variety of biologically active substances, including proteins, mRNA, miRNA, DNA, carbohydrates, and lipids. These vesicles act as carriers for their payload when transported horizontally across cells (Sultan et al., 2021). In 2013, MEVs in mouse stools were identified by Kang et al. (2013). They demonstrated how the stool MEVs from an IBD mouse model displayed substantial dysbiosis in comparison to the difference in the microbiota composition between the inflammatory and control phenotypes. This study demonstrates that EVs play a regulating function in intestinal immunity and homeostasis, even though it was unclear whether the dysbiosis was an effect of the inflammation or its cause. For instance, mice were prevented from developing colitis by the EVs of the gut bacterium *Akkermansia muciniphila*, and the proinflammatory cytokine IL6 was reduced in response to *E. coli* treatment (Kang et al., 2013). Clinical research has shed light on how the microbiome affects immunity and a variety of disorders. For instance, *Bacteroides thetaiotaomicron* EVs contain hydrolytic enzymes that, when shared with bacteria lacking hydrolytic enzymes, boost the potential digestion of gut microbiota. Therefore, administering EVs produced from particular bacterial strains may alter host nutrition, immunological signaling pathways, and the generation of bacterial metabolites. The EV-based network most likely represents significant links that organize the gut microbiota’s ecological units (Badi et al., 2017).

## 2 Small RNA and its biological function

### 2.1 Eukaryotic sRNA

Non-coding RNAs (ncRNAs) are a large group of RNA molecules that cannot encode proteins. ncRNA comprise 98% of all transcriptional output, which can be divided into two main subgroups: housekeeping ncRNA and short (less than 200 nt) and long (more than 200 nt) ncRNA (Wang and Li, 2012). Small non-coding RNAs (sncRNAs) are key mediators of post-transcriptional regulators in bacteria and eukaryotes that control gene expression through a variety of mechanisms (Wang and Fu, 2019). These include micro-RNAs (miRNAs), small interfering RNAs (siRNAs), Piwi-interacting RNAs (piRNAs) (Orendain-Jaime et al., 2021), and tRNA-derived small RNAs (tsRNAs) (Li et al., 2018). Apart from these, which mostly act as silencers, in 2006 Long-Cheng Li and colleagues identified small RNAs that target gene promoter sequences to activate expression in a process called RNA activation (RNAa) (Li et al., 2006).

#### 2.1.1 miRNA

miRNA is an endogenous sRNA with a length of about 22 nt (Bhaskaran and Mohan, 2014), which was first discovered by Lee and colleagues in the nematode *Caenorhabditis elegans* (Lee et al., 1993). They have the role of post-transcriptional regulators of gene expression and often lead to changes or prevent the production of protein products through binding to the complementary sequence of mRNA and interfering with the translation machines (Bhaskaran and Mohan, 2014. It should be noted that miRNAs, in addition to mRNA degradation, transcription silencing, and post-transcriptional silencing, play an important role in humans in modulating the process of apoptosis (Allmer and Yousef, 2014) and inhibiting the proliferation and migration of cancer cells (Ding et al., 2021). It also serves as diagnostic and prognostic biomarkers for diseases such as cancer (Saldanha et al., 2016; Liu et al., 2017; Yang et al., 2017), neurological disorders (Khoo et al., 2012), and type 2 diabetes (Mahjoob et al., 2022). In general, miRNAs in cancer can be classified into two categories: oncogenic (oncomiRs) and tumor suppressor (tsmiRs), which play a role in cancer progression and suppression, respectively (Wang and Li, 2012).

#### 2.1.2 siRNA

siRNAs are a class of 22 nt double-stranded RNAs (dsRNAs) that are produced endogenously or synthetically and are known as activators of the RNA interference (RNAi) mechanism (Wang and Li, 2012). In 2006, Fire and Mello won the Nobel Prize for coining the term “RNA interference” and discovering the mechanics of its occurrence. They reported that dsRNAs induce gene silencing through Watson-Crick base pairing with a complementary sequence in mRNAs (Alshaer et al., 2021). This makes siRNAs superior to monoclonal antibody drugs and small molecule therapies, because theoretically, any gene of interest can be targeted with the help of siRNA, while these drugs must recognize the complex spatial composition of specific proteins (Hu et al., 2020). Considering that, many human diseases are caused by excessive production of specific gene products such as oncogenes, siRNAs can be used to target the active genes. To date, many siRNAs have been used to treat ocular (Nikam and Gore, 2018), liver (Zhao et al., 2019), kidney (Stokman et al., 2010), cancer (Hou et al., 2006), etc., and are modified with various chemical compounds, and are phosphonated for proper function.

#### 2.1.3 piRNA

piRNA is an endogenous single-stranded sRNA with a length of ~27nt found in vertebrates and invertebrates (Wang and Li, 2012). The *PIWI* as a gene encoding piRNA was first identified in Drosophila in 1997, where mutants showed defects in germ cell maintenance (Cox et al., 1998). Due to the larger size of piRNAs compared to miRNAs, they bind to mRNA more tightly and it is thought that they can inhibit protein synthesis (Kamenova et al., 2023). Although the function of piRNA in humans is not fully understood, abnormal expression of Hiwi (ortholog humans Piwi) has been reported in a variety of cancers (Wang and Li, 2012). piRNAs have been identified as new prognostic and diagnostic tools for cancer (Guo et al., 2020) and MS (Kamenova et al., 2023).

#### 2.1.4 tsRNA

tRNAs comprise about 4-10% of all cellular RNAs, which are the most abundant type of small noncoding RNAs (sncRNAs). They are key components in the translation process and transport amino acids to the ribosome. Many studies have shown that many sncRNAs are derived from tRNAs that are ~15-50 nt in length (Watson et al., 2019) and play different roles in epigenetic regulation, gene expression, protein translation, and immune processes. tsRNAs are divided into two main types based on the length and site of tRNA cleavage, tRNA stress-induced RNA (tiRNA) and tRNA-derived fragment (tRF). Abnormal levels of tsRNAs have been reported in infectious, neurological, acquired metabolic diseases and cancer in humans. Researchers have examined the level of tsRNAs in serum as a biomarker in disease diagnosis (S. Li et al., 2018).

#### 2.1.5 saRNA

saRNA is a class of 21nt long sncRNAs that have the same structure and chemical components as siRNA, although their biological functions are opposite (Kwok et al., 2019). In 2000, saRNAs were first identified by Li et al., who reported that saRNAs activate gene expression in mammalian cells by targeting gene promoters (Li et al., 2006). Considering that saRNA is a very powerful tool for gene activation, it has been used in regenerative medicine and it is predicted that it is possible to combine saRNA with other drugs to improve treatment (Kwok et al., 2019). In overall, in various reports, sRNAs have been used as non-invasive clinical diagnostic biomarkers for diseases such as acute myeloid leukemia (Xia et al., 2023), neurodegenerative disease (Watson et al., 2019), renal cell carcinoma (Ding et al., 2021), and multiple sclerosis (Piket et al., 2019).

### 2.2 Bacterial sRNA

Bacterial sRNAs are transcribed from the intergenic regions of the bacterial genome, which are usually 50-400 nt in length (Wang and Fu, 2019) and are recognized as important elements of bacterial adaptation to environmental changes (Jørgensen et al., 2020). As bacteria are exposed to environmental fluctuations such as changes in pH, temperature, nutrient concentration, water and others, they use fast and flexible mechanisms for survival and reproduction (Raina et al., 2022). Bacterial sRNAs regulate various biological processes such as energy metabolism, quorum sensing (QS), biofilm formation, and stress response (Michaux et al., 2014). Gram-negative bacteria have unique structural features that require maintaining homeostasis in the inner membrane (IM), outer membrane (OM), and periplasmic space for microbial growth and cell proliferation. These cells use various stress response systems to monitor the status of membrane proteins, with sRNAs playing an essential role (Papenfort and Melamed, 2023). In addition, sRNAs are involved in modulating the stability or translation of mRNAs through short-base pair interactions and are among the main post-transcriptional regulators in bacteria (Raina et al., 2022). Here, pathways for bacterial metabolism and homeostasis are summarized, and specific roles for sRNA in the regulation of those pathways are presented in **Table 1**.

**Table 1:** sRNAs function in gram negative bacteria.

Role of sRNA		*Bacteria*	sRNA	Function	Ref
**Cellular metabolism**	Carbon metabolism	*E.coli*	SgrS Spot 42 GlmYZ	- Controlling the uptake and secretion of various carbohydrates - A global regulator of secondary carbon metabolism - Involved in cellular response to DNA damage - Glucosamine-6-phosphate synthetase (GlmS) expression control in response to its product (GlcN-6-P)	(Bobrovskyy and Vanderpool, 2014; Prasse and Schmitz, 2018); (De Lay and Gottesman, 2009; Prasse and Schmitz, 2018; Zhang et al., 2019) (Fröhlich and Gottesman, 2018; Prasse and Schmitz, 2018; Jørgensen et al., 2020) (Chareyre and Mandin, 2018; Fröhlich and Gottesman, 2018)
	Nitrogen metabolism	*E.coli P. stutzeri A1501*	CyaR SdsN NfiS	- A posttranscriptional repressor - Transcription regulator crp - Suppression of genes involved in the metabolism of oxidized nitrogen compounds - Optimization of nitrogen fixation through base pairing with the nitrogenase nifK mRNA	
	Amino acid metabolism	*P. aeruginosa PAO1 E.coli/Salmonella E.coli*	NalA NrsZ GcvB Sr1	- RNA leader-mediated antitermination- Translation activation- Prevent nitrogen starvation - Translation inhibition- Represses peptide transporters- Translation activation- Transcription regulator of arginine metabolism	
	Iron homeostasis regulation	*E.coli V. cholera S. dysenteriae P. aeruginosa Klebsiella pneumonia A. vinelandii*	RyhB RyhBRyhB PrrF1/PrrF2PrrHRyhB1/RyhB2ArrF	- Inhibiting the synthesis of unnecessary iron binding proteins (in iron deficiency conditions)- Overcome stress by increasing available iron- Controlling iron homeostasis genes - Directly involved in pathogenesis- Activation/suppression of pathogenicity during the course of infection by iron- Rapid degradation of iron-binding mRNAs- Regulation of biosynthesis genes - Involved in the capsule and acquisition of iron- Regulation of genes encoding iron-containing proteins	
**Quorum sensing and biofilm formation**	QSandBiofilm formation	*E.coli P. aeruginosa S. dysenteriae/Vibrio cholerae Vibrio cholerae*	MicA/McaSCsrB/CFimR2 PrrF RyhB Qrr1–5	- A post-transcriptional regulator of the OM protein- Increased swimming mobility- Regulation of cellular processes, such as biofilm formation and motility- Biofilm formation as a dominant mode of survival under nutrient depletion conditions- Control over motility and biofilm formation - Regulate quorum sensing by preventing the degradation of anthranilate - Control of genes involved in motility and biofilm formation- Increased virulence and biofilm formation through regulation of s at least four mRNAs (luxO/U, hapR, aphA and vca0939)	(Bak et al., 2015; Chareyre and Mandin, 2018; Huber et al., 2022; Raad et al., 2022)
**Stress response**	Acid stress response	*E.coli E.coli/ S. Typhimurium*	GadYDsrA/ArcZ/RprA6S RNA	- Regulation of acid stress tolerance- Stabilization of rpoS mRNA secondary structure- Increasing the response of bacteria to different stresses- Increasing the ability to survive under acid stress	(Ren et al., 2017; Lin et al., 2021); (Hu et al., 2019; Zhang et al., 2019); (Fröhlich et al., 2012; Bobrovskyy and Vanderpool, 2014; Negrete and Shiloach, 2017)
	Oxidative stress response	*E. coli P. stutzeri A1501 E.coli*	OxySNfiS NfiS	- Inhibits rpoS translation- Cell cycle arrest to allow DNA damage repair- Acts as a regulator- Integrates adaptation to H2O2 with other cellular stress responses- Help protect cells from oxidative damage- Increased tolerance to oxidative and osmotic stress	
	Osmotic stressand anaerobic growth condition	*E.coli*	RprAMicFMicA/RybBFnrS/ArcZ	- Activation of translation of rpoS mRNA- Repression of translation and stability of porin OmpF- Expressed under conditions of oxygen limitation	
	Phospho-sugar stress	*E.coli/Salmonella*	SgrS	- Changes in mRNA translation and stability-Production of SgrT and inhibition of major glucose transporter activity-Reducing the accumulation of phosphorylated sugars and stress-Promote growth	

Cellular metabolism is essential in bacteria for optimizing nutrient utilization. Regulatory mechanisms ensure that bacteria preferentially use the most favorable carbon, nitrogen, and amino acid sources based on their availability, energy efficiency, and growth requirements. sRNAs regulate metabolic processes by influencing metabolic enzymes, transporters and regulators. (Michaux et al., 2014). Carbon metabolism is a versatile and essential process that helps bacteria use organic compounds for energy production and the biosynthesis of nucleic acids, amino acids, lipids, and carbohydrates (Noor et al., 2010). Nitrogen metabolism enables bacteria to utilize nitrogen in different forms such as ammonium, nitrate, and amino acids, and use them to synthesize nitrogen-containing compounds necessary for cell. Amino acids metabolism includes the synthesis, breakdown, and conversion of amino acids, which are essential for the adequate supply of these building blocks for protein synthesis and carrying out cellular processes (Prasse and Schmitz, 2018).

Iron plays a dual role in bacteria, serving as a crucial micronutrient for bacterial growth while also potentially harming bacterial cells by generating reactive oxygen species (ROS) during aerobic metabolism. Therefore, iron homeostasis is critical for bacterial survival, and bacteria use complex mechanisms to absorb, store, and utilize iron (Klebba et al., 2021). Bacterial sRNAs play a crucial role in these metabolic processes as post-transcriptional regulators, influencing gene expression and metabolic pathways (Papenfort and Melamed, 2023). Quorum sensing (QS) is a cellular communication mechanism in bacteria and fungi that indicates population density and plays an important role in pathogenicity and biofilm formation (Michaux et al., 2014). When the concentration of signaling molecules reaches a certain threshold and binds to receptor proteins, it triggers the activation of genes that promote biofilm formation (Williams, 2007). The primary function of QS is to enable bacteria to monitor their environment and adjust their behavior accordingly. As a result, it allows bacteria to assess the number of neighboring bacteria and coordinate their activities as a collective group (Wang et al., 2022). Bacterial sRNAs are responsible for transcription regulation, biofilm formation and information integration by QS systems (Michaux et al., 2014).

In the natural habitat, bacteria face various stressors such as temperature and pH fluctuations, nutrient limitation, exposure to toxins, oxidative stress, and physical damage. Stress response mechanisms enable bacteria to sense and respond to these stressors and increase their survival. sRNAs play an essential role in coordinating the stress response by regulating the expression of stress-related genes, thereby enabling the activation or suppression of stress response pathways to support bacterial survival (Holmqvist and Wagner, 2017). When bacteria are exposed to acidic conditions as a result of metabolic processes, acid stress is induced. This in turn triggers the activation of bacteria’s acid resistance systems to protect against cell damage and maintain homeostasis (Dawan and Ahn, 2022).

ROS are released upon exposure to oxygen or as byproducts of metabolic reactions. In these conditions, bacteria use antioxidant defense systems (such as catalase and superoxide dismutase enzymes and small molecule antioxidants) to neutralize ROS and protect cellular components against oxidative damage (Seixas et al., 2022). When the osmolarity of the surrounding environment changes, it leads to a change in water availability and ion concentration, which bacteria maintain osmotic balance through the synthesis and absorption of compatible solutes and prevent cell shrinkage or lysis (Krämer, 2010). Phosphosugar stress arises from an imbalance in the availability or use of phosphosugars, which are crucial for cellular processes like energy metabolism and cell wall biosynthesis. Bacteria use regulatory proteins and metabolic pathways to adapt and restore phospho-sugar homeostasis (Papenfort et al., 2013). Overall, sRNAs are a flexible and rapid tool for gene regulation in bacteria, allowing them to respond to changing environmental conditions and optimize their survival strategies (Papenfort and Melamed, 2023).

### 2.3 sRNA applications

Natural or synthesized sRNAs are used to silence or regulate gene expression related to disease pathways, after identifying the specific mRNA involved in the pathway. sRNAs complementary to target mRNA sequences are designed or identified to inhibit their activity. Using chemical modifications such as locked nucleic acids (LNAs) or phosphorothioate linkages increases the stability and protects sRNAs from degradation by cellular nucleases Finally, efficient delivery systems are used to deliver engineered sRNAs (Rupaimoole and Slack, 2017). For example, in a study, a new sRNA called EsrF was reported that through binding to flhB mRNA leads to increased bacterial motility and adhesion to host cells, which is ultimately associated with increased infection. Engineered sRNA is predicted to reduce infection by not binding to mRNA (Jia et al., 2021).

## 3 Bacterial outer membrane vesicles

Gram-negative bacteria produce ectosomes called outer membrane vesicles (OMVs), which are spherical lipid bilayer structures with sizes ranging from approximately 20 to 250 nm (Yang et al., 2018). OMVs are present in all stages of bacterial growth, and they represent the structure of the bacterial OM (Furuyama and Sircili, 2021). In 1967, Chatterjee and Das first identified OMVs during the natural growth of *Vibrio cholera* (Chatterjee, 1967. After that, the presence of OMVs was observed in different types of Gram-negative bacteria (Devoe and Gilchrist, 1973) and even in patients with meningococcal infection, which indicates the role of OMVs in bacterial pathogenesis (DeVoe and Gilchrist, 1975). The structure of OMVs allows them, in addition to carrying different cargoes (lipopolysaccharides (LPS), phospholipids, peptidoglycan (PG), proteins, nucleic acids, etc.), to be genetically engineered and chemically modified to increase efficiency (Xue et al., 2022).

**Figure 1:**
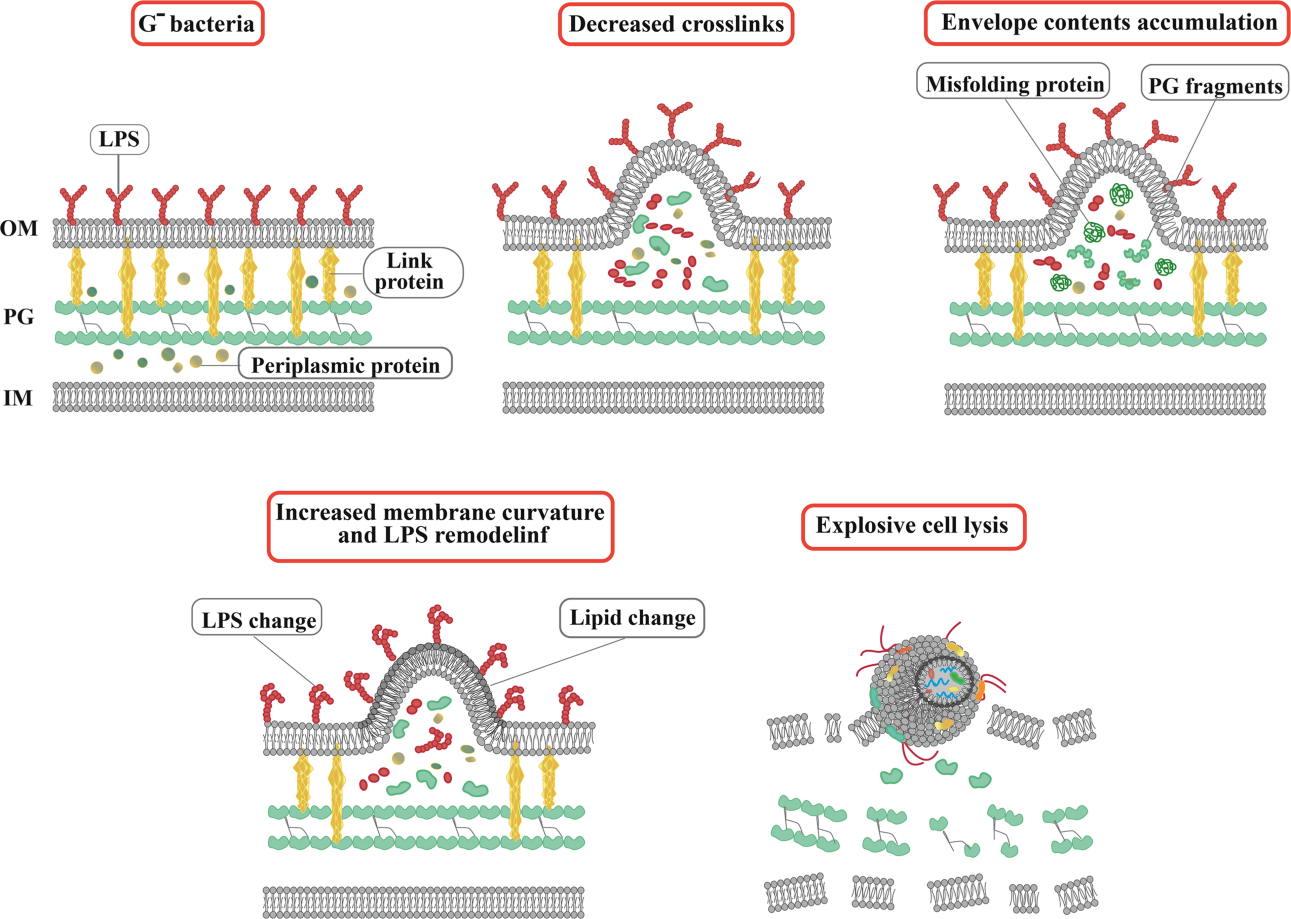
Actual and schematic structure of OMVs. Steps of OMVs biogenesis in Gram-negative bacteria. The arrows indicate OMVs. SEM, scanning electron microscope; TEM, transmission electron microscopy.

Bacterial extracellular vesicles contain a significant amount of RNA, and among mRNA, tRNA, rRNA, and sRNA, sRNAs occupy a significant part (Luo et al., 2023). In the Gram-negative bacteria, which are characterized by an OM rich in lipopolysaccharide outside the thin layer of PG, the formation of OMVs is a highly spontaneous and conserved process (Xue et al., 2022). In general, the biogenesis of OMVs is based on three mechanisms (**Figure 1**): 1) Reducing the interaction between the OM and the underlying structures such as the PG layer and its associated lipoproteins (Pita et al., 2020). 2) Accumulating misfolded proteins, PG fragments, and LPS in a specific region of the bacterial periplasm, leading to deformation of the upper OM. 3) Altering membrane chemical-physical properties and asymmetric distribution of phospholipids, which modulates asymmetric membrane expansion, protrusion, and OMV biogenesis (Luo et al., 2023).

In addition to OMVs, membrane vesicles of Gram-negative bacteria can also be divided into outer-inner membrane vesicles (OIMVs) and explosive outer membrane vesicles (EOMVs) based on their formation pathways, structure, and composition (Toyofuku et al., 2019). In general, three functions have been considered for OMVs, including, bacterial survival (nutrient acquisition, stress response), regulation of interactions in bacterial communities (biofilm, quorum sensing), and induction of pathogenesis (immunomodulation) (Diallo et al., 2022). OMVs, as protectors and carriers of functional and gene-regulatory sRNAs, mediate direct contact-free transfer of sRNA fragments between bacterial and mammalian cells (Han et al., 2022). sRNAs in OMVs act as interspecies communication molecules to modulate gene expression in different cell types and species (Li et al., 2020).

### 3.1 Bacterial-derived nanoparticle

Nanoparticles (NP) have many potential properties that promote their use in various biomedical applications including diagnostics, cellular imaging, chemical assays, drug delivery and therapy. However, NPs have limitations such as cytotoxicity, low immunogenicity, low cellular uptake by target cells, non-selective targeting and increased clearance rate, which make their use challenging (Ajam-Hosseini et al., 2023). OMVs have gained attention by overcoming these limitations and are valuable to researchers in biomedical applications (Naskar et al., 2021). The use of bacterial OMVs as a therapeutic strategy to overcome the challenges of biocompatibility and large-scale production associated with synthetic nanocarriers has been made possible by advances in genetic engineering. Bacterial OMVs have a tough membrane that provides stability and reduces leakage into the systemic circulation (Gujrati et al., 2019), such that approximately 75% of the surface of *E. coli* is occupied by rigid lipopolysaccharides (Magennis et al., 2014).

OMVs have shown great potential in biomedical applications as they are used as nanocarriers for drug delivery, bioimaging, immune system modulation (Naskar et al., 2021) and various therapeutic strategies, including vaccine development 48, gene therapy, and cancer treatment (Pan et al., 2022). With the help of genetic engineering, membrane modification, and membrane coating bacteria can be manipulated as nanovesicles with non-toxic OM components (Liu L. et al., 2020). Genetic engineering is an ideal tool for designing nanoplatforms related to bacteria in the treatment of various diseases with the help of transferring drugs, genes, proteins, and enzymes.

In this regard, Gujrati et al. bioengineered *E.coli* to produce OMVs with less toxicity to deliver siRNA and antitumor drug melanin (Gujrati et al., 2019). Compared to Gram-positive bacteria, Gram-negative bacteria are easier to bioengineer, so most studies have focused on them (Liu L. et al., 2020). Some researchers have studied the genetic modification of bacterial protoplasts because they are easily manipulated to make nanovesicles with non-toxic OM components. Therefore, bacterial protoplast-derived nanovesicles (PDNVs) have been used for adjuvant-free vaccine delivery in bacterial infection, showing remarkable efficacy and safety (Kim et al., 2015). Coating NPs with bacterial OMVs (OMV-NPs) is a facile process that has good biocompatibility and alleviates some of the limitations of traditional surface modifications (Naskar et al., 2021). Due to the large number of immunogenic antigens and different pathogenic molecular patterns in the bacterial membrane that play an important role in creating innate and adaptive immunity, it makes them desirable in various research fields (Liu W. L. et al., 2020). By modifying properties of bacteria such as membrane proteins (Anwar et al., 2021), lipid compositions (Pichler and Emmerstorfer-Augustin, 2018), the inflammatory responses of the body against them and side effects can be reduced (Cao et al., 2019). Although, bacteria-derived nanoparticles have created a new frontier in medical treatment strategies, the use of sRNA encapsulated with OMV-NPs requires further investigation.

### 3.2 OMVs engineering

In therapeutic strategies, OMVs are engineered through immune modulation, size, surface modification and composition. Since OMVs have intrinsic immunomodulatory properties, it is possible to modulate the immune response for therapeutic purposes by modifying compounds such as immunostimulatory or immunosuppressive molecules (Kim et al., 2013). OMV size can be controlled during purification and can impact their stability, biodistribution, and cellular uptake. OMVs’ efficiency in binding to target cells can be improved by using specific ligands and peptides. Furthermore, genetic modification of the original bacterial strain can introduce the desired surface proteins. It should be noted that the engineering of OMVs is different based on the desired therapeutic application and desired cargo (Huang et al., 2022).

## 4 Clinical relevance of sRNA encapsulated in OMVs derived from Gram-negative bacteria

**Figure 2:**
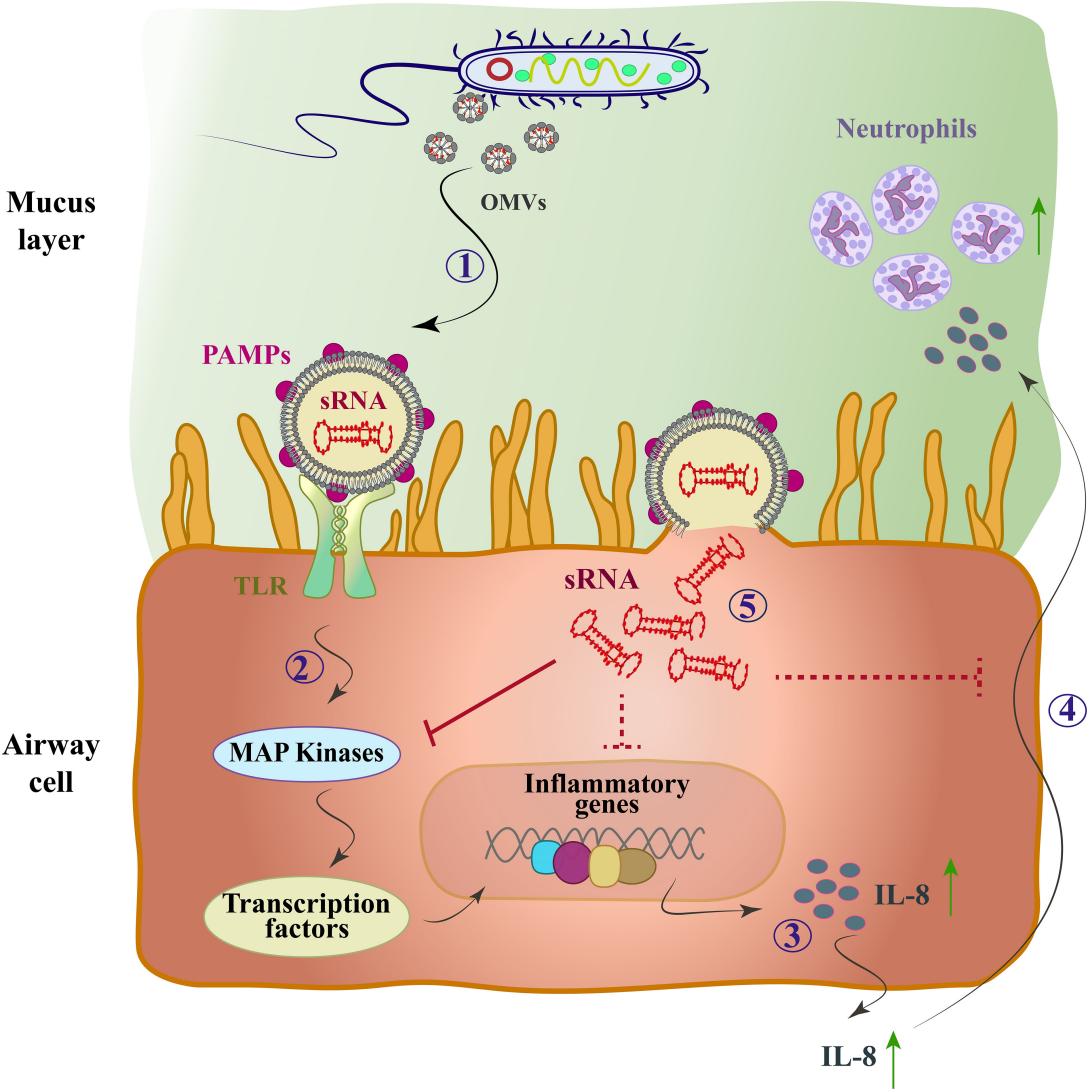
The mode of action of the OMV-encapsulated sRNA of *P. aeruginosa* in human airway epithelial cells. *P. aeruginosa* produces OMVs after entering the mucosal layer of the airways. (1) OMVs bind to TLRs through PAMPs. (2) The MAP-kinase (TLR/MAPK) signaling pathway is activated and induces the host’s innate immune response. (3) Transcription factors are activated and cause up-regulation of IL-8 mRNA and IL-8 secretion (4) IL-8 attracts neutrophils and they phagocytose *P. aeruginosa* by entering the lungs. (5) OMVs fuse with sRNA and enter cells, which by targeting mRNA of the MAPK signaling pathway upstream of IL-8 leads to reduced host IL-8 secretion and neutrophil recruitment. Solid arrows, direct inhibitor; Dashed arrows, indirect inhibitor.

Due to the limited information available regarding the biological role of bacterial sRNAs within OMVs, few studies have been conducted so far. However, functional sRNAs derived from bacterial OMVs have been identified using high-throughput RNA sequencing. Recent studies have investigated the potential role of vesicular and bacterial sRNAs in host-pathogen interactions. For the first time, Koeppen et al. delivered sRNAs into host cells using OMVs from *Pseudomonas aeruginosa* bacteria (Koeppen et al., 2016). *P. aeruginosa* is a Gram-negative bacterium that is the main cause of bacterial colonization in chronic obstructive pulmonary disease (COPD) affected patients (Nakamoto et al., 2019). They also showed that the actual expression level of sRNAs in OMVs can lead to a decrease in host immune response by reducing cytokine secretion. The pathogen-associated molecular patterns (PAMP) on the outside of OMVs induce a proinflammatory host immune response (Koeppen et al., 2016). PAMPs, which includes LPS, peptidoglycan, flagellin, lipoproteins, and purines, bind to toll-like receptors (TLR) of host airway epithelial cells and lead to increased secretion of pro-inflammatory cytokines, especially IL-8, through the mitogen-activated protein kinase (MAPK) signaling pathway (Bauman and Kuehn, 2006). Cytokine secretion recruits and activates neutrophils to enhance the clearance of *P. aeruginosa* infection, and the increase of IL-8, a potent chemoattractant for neutrophils, leads to extensive neutrophil infiltration and the production of proteolytic enzymes such as elastase, leading to bacterial phagocytosing and tissue destruction (Huang et al., 2020) (**Figure 2**). In other words, sRNAs reduced OMV-stimulated IL-8 secretion by translocation from OMVs to host cells (Zhang et al., 2020).

*P. aeruginosa* has received much attention as the first virus to use OMV-encapsulated sRNAs to suppress the host cell immune response, so we investigated it for this purpose. In **Table 2**, we reviewed more examples of the current application of OMV-encapsulated sRNAs of Gram-negative bacteria for biomedical purposes. Although, study of these processes remains complex and challenging and requires further investigations in the field of transmission mechanism, they provide a new model on host-pathogen dynamics.

**Table 2:** Description of secreted sRNAs by Gram-negative bacterial and their clinical relevance.

Gram-negative bacterial	Cargo	Target cell	Functions	Ref
*Escherichia coli* BL21 (ΔmsbB) Mutant *E. coli*	tRNALys-pre- miRNA-126 siRNA siRNA	Breast cancer cell Tumor cells Kinesin spindle protein	- Inhibits cell proliferation- Target gene expression dropped in response to invading miRNA- Accumulation within tumor tissues- Inhibiting the growth of tumors - Significantly accelerated tumor development- *In vivo* inhibition by targeted gene silencing - OMVs can be utilized to deliver drugs to targeted cancer cells.	(Aytar Çelik et al., 2023); (Kuerban et al., 2020); (Jalalifar et al., 2023)
*Pseudomonas aeruginosa*	sRNA52320	Airway epithelial cells	- Decreased LPS- and OMV-induced IL-8 secretion by cultured primary human airway epithelial cells.	(Koeppen et al., 2016)
*Klebsiella pneumoniae*	NA	HEp-2 cells	- Raised IL-1b and IL-8(Intertracheal challenge in neutropenic mice as a model)	(Mehanny et al., 2021)
*Helicobacter pylori*	sncRNA	gastric adenocarcinoma cell	- It has been discovered that the functions of sncRNAs sR2509025 and sR989262, which interact with host cells via OMV secretion and inhibit the secretion of interleukin 8 (IL-8), which targets mRNAs encoding multiple kinases in the LPS-stimulated mitogen-activated protein kinase (MAPK) signaling pathway, have not been fully elucidated.	(Jalalifar et al., 2023)
*Aggregatibacter actinomycetemcomitans*	sRNA	Jurkat T-cells Human macrophage-like cellsMacrophage	- IL-5, IL-13, and IL-15 anti-inflammatory cytokines are decreased *in vitro* by msRNA. - Microbiological EV-derived small seRNAs’ cytoplasmic transport and activity in macrophages.- Increased TNF-production through the NF-B and TLR-8 signaling pathways.- TLR-8 and NF-kB signaling pathways were used by exRNAs to enhance the generation of TNF-a.	(Pita et al., 2020) (Han et al., 2019)
*Porphyromonas gingivalis* (ATCC 33277)	sRNA	Jurkat T-cells,	- IL-5, IL-13, and IL-15 anti-inflammatory cytokines are decreased *in vitro* by msRNA. - OMVs are able to penetrate host cells.- RNA transmission between *P. gingivalis* strains via vesicles.	(Zou et al., 2023)
*B. fragilis*	RNA	Intestinal epithelial cells	- Greater activation of innate immune receptors in the host - Toll-like receptors (TLR)-2, -4, -7, and nucleotide-binding oligomerization domain-containing protein 1 (NOD1) in the host are activated.	(Gilmore et al., 2022)
*Salmonella enterica Salmonella* sp.	sRNA NA	HeLa cells Dendritic cells (MOUSE)	- Regulate JAK-STAT signaling in host cells. - Actively influence both common and disparate host pathways to successfully infect the host. - Increased MHC-II and CD86 expression - Increased TNF and IL-12 release.	(Lee, 2019)	(Westermann et al., 2016) (Mehanny et al., 2021)

NA, Not available.

## 5 A summary of research challenges

Although OMVs-encapsulated sRNAs have been widely used in research, there are still many challenges and gaps in this field that limit the practical applications of OMVs, including:

### 5.1 OMV toxicity

There are two main strategies to reduce LPS-induced OMV toxicity. 1) Modifying the genes responsible for LPS synthesis (msbA_2_, msbB, lpxL1, lpxM) in order to reduce the number of acyl chains or phosphate groups of LPS, which leads to the detoxification of molecules in bacteria (Simpson and Trent, 2019). However, there are G^-^ mutant bacteria such as *E. coli* EMKV15 that do not contain LPS and may be a better choice for drug delivery (Liu et al., 2022). 2) Chemical modification of LPS species from *Salmonella minnesota* to produce a mixture of mono-phosphorylated lipid A species (MPL) with less toxicity as reported in 1982 by Riby et al. (Qureshi et al., 1982). In 2009, following FDA approval, mainly 3-O-deacyl-4’-monophosphoryl lipid A became an adjuvant in MPL vaccines (Casella and Mitchell, 2008).

### 5.2 Ambiguous mechanism

Although engineered BEVs can serve as a targeted drug delivery system, further research is still needed for more precise performance with fewer side effects (Gujrati et al., 2014). BEVs are internalized through various mechanisms such as clathrin/non-clathrin-mediated endocytosis, micropinocytosis, and membrane fusion (Liu et al., 2022). For example, the size of *H. pylori* OMVs can determine the host cell entry mechanism to be taken up by gastric epithelial cells through clathrin-mediated endocytosis and lipid raft-mediated endocytosis. *Burkholderia pseudomallei* is the causative agent of melioidosis, a severe infectious disease in which EVs are taken up by host cells through various mechanisms including clathrin-mediated endocytosis, macropinocytosis, and phagocytosis (Li et al., 2020).

OMVs from *Legionella pneumophila* can deliver contents to the host cell membrane by fusing with eukaryotic membrane systems. Membrane properties and the exact mode of interaction between *L. pneumophila* OMVs and host cell surfaces are not fully understood. Adhesion to plasma membrane proteins and subsequent uptake by phagocytosis are predicted to be effective (Jäger et al., 2015). These examples demonstrate that the effectiveness of these uptake mechanisms can vary depending on the characteristics of BEVs and the receptor cell type, which is crucial for their manipulation in research.

### 5.3 Heterogeneous contents

BEVs are rich in biological and functional molecules whose research field is still evolving. The specific cargo of BEVs can vary depending on factors such as the bacterial strain, environmental and host conditions, not all of which may be favorable for internalization into recipient cells (Taboada et al., 2019). This variability provides valuable insights into their functional roles and potential impact on host-microbe interactions.

### 5.4 Complicated isolation and purification

Presented BEV separation and purification methods, such as ultracentrifugation, are time-consuming and expensive and require advanced equipment. It also usually results in low yield due to loss of desired products (Huang et al., 2022). On the other hand, due to insufficient knowledge about specific (surface) markers in BEVs, the discovery of specific markers can be one of the most promising methods (Liu et al., 2022). Thus, new methods such as membrane bioreactors with separation functions and OMVs purification methods based on reversible phase separation, can be utilized to address these issues (Huang et al., 2022).

Additionally, there may be potential negative consequences associated with the use of OMV-encapsulated bacterial sRNA in treatment, such as unintended gene silencing or dysregulation in non-target cells or tissues (Choi et al., 2017).

Since OMVs originate from bacteria, they can also trigger immune responses that lead to inflammation or immunogenic reactions. Prolonged exposure to OMV-encapsulated sRNA may result in immune-related toxicities. Finally, the long-term effects of this treatment could potentially cause genomic instability or alterations in cellular processes (Kim et al., 2013).

## 6 Conclusion

New drug delivery systems use nanotechnology, biomaterials, and RNA to improve drug stability, increase targeting capabilities, and controlled release. Compared to conventional drug delivery methods, nanotechnology-based systems offer advantages such as controlled release, increased tissue penetration, and targeted delivery to specific cells or tissues (Ajam-Hosseini et al., 2023). Drug delivery systems based on biological materials such as hydrogels, microparticles, and scaffolds can improve the sustained release of drugs, local delivery, and therapeutic outcomes. This system has the potential to overcome limitations associated with conventional methods, such as repeated dosing and systemic side effects (Sun et al., 2023).

RNA-based therapies, including mRNA and small interfering RNA (siRNA), have attracted considerable attention in recent years as they enable modulation of gene expression compared to conventional methods and offer opportunities for personalized medicine (Kwok et al., 2019). Significant structural investigations have been conducted on RNA motifs and/or RNA-protein complexes, which aid drug development through structure-based virtual screening (Yang et al., 2023). The effectiveness of these new systems depends on the specific application, the nature of the drug, and the disease or condition in question.

For this purpose, this review provides sufficient insight into the clinical significance of Gram-negative bacterial sRNAs in biomedical applications. However, more studies are needed to solve the mentioned research challenges and use the structure of Gram-negative bacterial OMVs to diagnose human diseases with robust methods and develop new clinical applications.

## Author contributions

MA-H: Writing – original draft. FA: Writing – original draft. FP: Writing – review & editing. HF: Writing – review & editing.
